# Insulin Resistance and Cardiovascular Risks in Different Groups of Hemodialysis Patients: A Multicenter Study

**DOI:** 10.1155/2019/1541593

**Published:** 2019-06-11

**Authors:** Tuyen Van Duong, Chun-Kuang Shih, Te-Chih Wong, Hsi-Hsien Chen, Tso-Hsiao Chen, Yung-Ho Hsu, Sheng-Jeng Peng, Ko-Lin Kuo, Hsiang-Chung Liu, En-Tzu Lin, Chien-Tien Su, Shwu-Huey Yang

**Affiliations:** ^1^School of Nutrition and Health Sciences, Taipei Medical University, Taipei, Taiwan; ^2^Department of Nutrition and Health Sciences, Chinese Culture University, Taipei, Taiwan; ^3^Department of Nephrology, Taipei Medical University Hospital, Taipei, Taiwan; ^4^School of Medicine, Taipei Medical University, Taipei, Taiwan; ^5^Department of Nephrology, Taipei Medical University- Wan Fang Hospital, Taipei, Taiwan; ^6^Division of Nephrology, Department of Internal Medicine, Taipei Medical University- Shuang Ho Hospital, Taipei, Taiwan; ^7^Division of Nephrology, Cathay General Hospital, Taipei, Taiwan; ^8^Division of Nephrology, Taipei Tzu-Chi Hospital, Taipei, Taiwan; ^9^Department of Nephrology, Wei Gong Memorial Hospital, Miaoli, Taiwan; ^10^Department of Nephrology, Lotung Poh-Ai Hospital, Yilan, Taiwan; ^11^School of Public Health, Taipei Medical University, Taipei, Taiwan; ^12^Department of Family Medicine, Taipei Medical University Hospital, Taipei, Taiwan; ^13^Research Center of Geriatric Nutrition, Taipei Medical University, Taipei, Taiwan; ^14^Nutrition Research Center, Taipei Medical University Hospital, Taipei, Taiwan

## Abstract

**Background:**

To investigate the association between insulin resistance (IR) and cardiovascular disease (CVD) risks among hemodialysis patients.

**Methods:**

We conducted a cross-sectional study between 2013 and 2017, on 384 hemodialysis patients from seven hospital-based-dialysis centers. HOMA-IR is classified according to median value. The CVD risks were defined by the K/DOQI Guidelines. Logistic regression analysis was used.

**Results:**

Patients' age was 60.9 ± 11.8, 58.1% men, and 40.3% overweight/obese. The median of HOMA-IR was 5.4, 82.8% high systolic blood pressure, and 85.7% hyperhomocysteinemia. In multivariate analysis, IR was significantly associated with higher odds of low high-density lipoprotein cholesterol, high triglyceride, and impaired fasting glucose in groups of normal weight, overweight/obese, nondiabetes, diabetes, and overall sample. IR linked with elevated high-sensitive C-reactive protein in normal weight patients (odd ratio, OR=2.21, 95% confidence interval, 1.16-4.22, p < .05), with hypoalbuminemia in normal weight patients (OR=8.31, 95% CI, 2.35-29.37, p < .01), in nondiabetes patients (OR=6.59, 95% CI, 1.81-23.95, p < .01), and overall sample (OR=3.07, 1.51-6.23, p < .01).

**Conclusions:**

The level of IR and prevalence of CVD risks were high in hemodialysis patients. IR was independently associated with CVD risks.

## 1. Introduction

The incidence and prevalence of treated end-stage renal disease (ESRD) have been steadily increased over the past decades across countries. In 2014, Taiwan has reported the highest number of treated ESRD with 455 new cases and the prevalence of 3219 patients per million general population (PMP). Taiwan has also experienced the highest number of maintenance hemodialysis in the world with 3093 patients PMP, 90% of them receiving in-center hemodialysis [1].

Cardiovascular disease (CVD) has been reported as the leading cause of death and disability all over the world. In 2013, CVD accounted for about 17 million deaths and 329 million disability adjusted life-years lost [2]. In ESRD patients, the cardiovascular cause of death is 10-20 times higher in the healthy population and accounted for more than half of all death [3].

Insulin resistance (IR) is with high prevalence in the ESRD patients [4]. IR its self is a risk for CVD and strongly associates with other CVD risks (dyslipidemia, hypertension, and inflammation) through several pathophysiologic mechanisms, which is well documented [5]. In ESRD patients undergoing hemodialysis, the cardiovascular risks worsen the arterial stiffness which contributes to the development of cardiovascular events and diseases [6]. IR is the anterior consequence of obesity [7]. In ESRD patients, IR then links to dyslipidemia, impaired fasting glucose, and cardiovascular risks and events [5, 8, 9]. In empirical researches, IR is closely associated with cardiovascular risks such as obesity, hypertension, and dyslipidemia [10], anemia [4], inflammation [11, 12], and echocardiography parameters [12]. In turn, IR significantly predicts cardiovascular diseases and mortality in ESRD patients [13–16]. Therefore, assessment of IR is critically important work for nephrologist and nurses to follow up patients and have appropriate interventions.

The ESRD has created a heavy burden for the healthcare system all around the world over the past decade [1]. However, the number of clinicians has not adequately increased to meet the greater demand for renal treatment [17]. The early detection of the IR and its associated factors might contribute to prevent CVD risks and reduce the burden. On the other hand, improving IR might be an important therapeutic target and contribute to better health outcomes in hemodialysis patients [18, 19]. This study was to assess the prevalence and explore the association between IR and CVD risk factors among ESRD patients undergoing hemodialysis.

## 2. Materials and Methods

### 2.1. Study Design

A clinical cross-sectional study was conducted between September 2013 and April 2017 in seven dialysis centers in Taiwan. A total of 384 hemodialysis patients were recruited from Taipei Medical University Hospital (55 patients collected from September to December 2013; 42 patients collected from November 2016 to January 2017); Taipei Medical University, Wan Fang Hospital (51 patients collected from April to May 2014); Taipei Medical University, Shuang Ho Hospital (39 patients collected in December 2014); Cathay General Hospital (41 patients collected in March 2016); Taipei Tzu-Chi Hospital (57 patients collected in November 2016); Wei-Gong Memorial Hospital (59 patients collected from February to March 2017); and Lotung Poh-Ai Hospital (50 patients collected in April 2017).

### 2.2. Hemodialysis Patients and Data Sources

We included patients aged above 20 years, receiving thrice-weekly hemodialysis treatment for at least 3 months and adequate dialysis quality (equilibrated Kt/V ≥ 1.2 g/kg/day). The exclusion criteria were patients who were diagnosed with pregnancy, amputation, hyperthyroidism, hypothyroidism, and malignancy, received tube feeding, exhibited hepatic failure or cancer, were hospitalized within one month prior to the recruitment, or were scheduled for surgery. Volume overload or edema closely linked with other clinical instability [20]. Therefore, patients with evidence of edema were excluded in the current study and in previous studies [21–23].

The eligible patients in selected hospitals signed the informed consents before conducting chart reviews and laboratory evaluations. The patients' medical records were reviewed. The blood samples were collected by licensed nurses, at the start of the first dialysis session of the week, and then analyzed in the hospital laboratory by using commercially available test kits, which was described carefully in previous studies [24, 25].

### 2.3. Insulin Resistance Index

The blood samples collected by the registered nurse were centrifuged in each hospital laboratory. The serum was separated and kept in the ice-pack, then sent to the laboratory in Taipei Medical University Hospital for serum insulin analysis. Therefore, all samples were analyzed with the same commercial kit. The homeostatic model assessment of insulin resistance index (HOMA-IR) is used to assess IR. The index is calculated using the formula developed by Matthews et al. [26]:

(1) HOMA-IR=fasting plasma  insulin  μU/mL×fasting plasma  glucose  mg/dL405.

Patients were separated into two groups based on the median value as the nonnormal distribution of HOMA-IR; this method was applied in previous studies [14, 27].

### 2.4. Cardiovascular Risks

The traditional and nontraditional CVD risks were described in the previous study [22] and the current study with the details below. In the present study, more factors were assessed and reported, such as physical activity, medical history (diabetes, hypertension, and cardiovascular disease), and other biochemical parameters (blood urea nitrogen, uric acid, creatinine, and fasting plasma insulin).

#### 2.4.1. Traditional CVD Risk Factors

The traditional risks of cardiovascular diseases include factors which were mentioned in previous studies [28, 29]. (1) Hypertension: systolic blood pressure (SBP) ≥ 130 mmHg and diastolic blood pressure (DBP) ≥ 85 mmHg [30]; (2) impaired fasting glucose (IFG): patients diagnosed with type 2 diabetes mellitus or fasting plasma glucose (FPG) ≥ 100 mg/dL [30]; (3) dyslipidemia including high serum triglyceride (TG) level at TG ≥150 mg/dL, low level of serum high-density lipoprotein cholesterol (HDL-C) at < 40 mg/dL in men and < 50 mg/dL in women, high level of serum low-density lipoprotein cholesterol (LDL-C) at ≥ 100 mg/dL, and high serum total cholesterol at TC ≥ 200 mg/dL [31].

#### 2.4.2. Nontraditional/Novel CVD Risk Factors

Anemia: the targeted hemoglobin (Hb) level should be 11g/dL or greater, as moderately strong recommended by The National Kidney Foundation Kidney Disease Outcomes Quality Initiative (K/DOQI) Work Group [32]. Anemia is classified as Hb < 11 g/dL. Mineral metabolism abnormalities: albumin-corrected calcium = total calcium (mg/dL) + 0.8 x (4.0 – serum albumin in g/dL) [33]. Corrected calcium and phosphate levels at each time were used to calculate calcium-phosphate product (Ca x PO_4_). The serum calcium (Ca) is classified into low level (Ca < 8.4 mg/dL), normal level (Ca 8.4- 9.5 mg/dL), and high (Ca > 9.5 mg/dL). The serum phosphate (PO_4_) is also classified into low level (PO4 < 3.5 mg/dL), normal (PO_4_ 3.5- 5.5 mg/dL), and high (PO4 > 5.5 mg/dL). Calcium-phosphate product is classified into normal (Ca x PO_4_ < 55 mg^2^ / dL^2^) and high (Ca x PO_4_ ≥ 55 mg^2^ / dL^2^). In addition, intact parathyroid hormone (iPTH) is classified as normal (iPTH 150-300 pg/mL), and high (iPTH ≥ 300 pg/mL) [34]. Hyperhomocysteinemia is defined as total plasma homocysteine (Hcy) > 14 *μ*mol/L [29]. Inflammation is defined as high sensitive C-reactive protein (hs-CRP) > 0.3 mg/dL as the risk factor for CVD [35]. The poor nutritional status is defined as serum albumin ≤ 3.5 mg/dL as applied in hemodialysis patients from 11 countries in the Dialysis Outcomes and Practice Patterns Study (DOPPS) [36]. Hyperkalemia is identified as serum potassium ≥ 5.0 mEq/L as the risk of cardiovascular mortality in hemodialysis patients [37].

### 2.5. Statistical Analysis

The descriptive analyses describe the patients' characteristics, insulin resistance (IR), cardiovascular disease risk factors via the mean, standard deviation, or median, interquartile range, frequency, and percentage. The independent-samples t-test, Chi-square test, and Mann-Whitney* U* test were used appropriately to test the distribution of patients' characteristics, CVD risks, and HOMA-IR in different groups of body mass index (BMI) and DM. In order to carefully examine the association between IR and traditional and nontraditional risk factors, the multivariate logistic regressions are used to estimate the odd ratios. Since obesity is the most common cause of IR [7], we analyzed the association in different groups of BMI (normal weight versus overweight/obese). The associations were also analyzed in a group of patients with diabetes and nondiabetes. The analyses were adjusted for age and gender, hemodialysis vintage, Charlson comorbidity index, and body mass index (for overall sample). These adjusted factors might be the confounders as they showed the relationship with IR [38–42]. All statistical analyses are performed by the SPSS for Windows v20.0 (IBM Corp., New York, USA). The significant level is set at p value < .05.

### 2.6. Ethical Approval

The study is approved by the Joint Institutional Review Board of Taipei Medical University (TMU-JIRB No. 201302024), which was for conducting the study in three hospitals of Taipei Medical University (Taipei Medical University Hospital, Wan-Fang Hospital, Shuang Ho Hospital), Wei-Gong Memorial Hospital, and Lotung Poh-Ai Hospital; the ethical committee of Cathay General Hospital (CGH-OP104001); and Taipei Tzu-Chi Hospital (04-M11-090). All patients involved in the study have signed the informed consent statement.

## 3. Results

Of the total sample, the average age of patients was 60.9 ± 11.8, 58.1% men, and 40.3% overweight or obese. The traditional CVD risk factors included high SBP (82.8%), high DBP (25.5%), high TC (16.7%), high LDL-C (48.4%), low HDL-C (65.9%), high TG (40.6%), and impaired fasting glucose (69.5%). The nontraditional CVD risks included anemia (58.3%), low calcium (8.3%), high calcium (35.2%), low phosphate (7.0%), high phosphate (35.4%), high calcium-phosphate product (25.5%), high intact parathyroid hormone (42.7%), hyperhomocysteinemia (85.7%), elevated hs-CRP (45.6%), and low serum albumin (12.0%; Table 1).

In comparison with normal weight patients, those with overweight/obese had a higher prevalence of low HDL-C, high TG, IFG, low serum Ca, elevated CaxPO_4_, elevated hs-CRP, and elevated HOMA-IR (p < .05). On the other hand, patients with DM had a higher proportion of high SBP, low HDL-C, high TG, IFG, elevated iPTH, low serum albumin, and elevated HOMA-IR, as compared with non-DM patients (p < .05; Table 1).

The results of multivariate regression analyses are shown in Tables 2 and 3. After control for age, gender, hemodialysis vintage, Charlson Comorbidity Index, and physical activity, in normal weight patients, IR was significantly associated with higher odds of low HDL-C (OR, 1.75, 95%CI, 1.01-3.06, p < .05), high TG (OR, 3.41, 95%CI, 1.78-6.53, p < .001), IFG (OR, 8.15, 95%CI, 4.14-16.02, p < .001), elevated hs-CRP (OR, 2.21, 95%CI, 1.16-4.22, p < .05), and lower odd of hypoalbuminemia (OR, 8.31, 95%CI, 2.35-29.37, p < .01), but with lower odds of high SBP (OR, 0.36, 95%CI, 0.17-0.76, p < .01), high PO4 (OR, 0.52, 95%CI, 0.28-0.96, p < .05), and hyperkalemia (OR, 0.39, 95%CI, 0.22-0.70, p < .01). In overweight/obese patients, IR was significantly linked with higher odds of low HDL-C (OR, 4.15, 95%CI, 1.71-10.06, p < .01), high TG (OR, 3.06, 95%CI, 1.53-6.13, p < .01), and IFG (OR, 10.76, 95%CI, 3.36-34.5, p < .001).

After being adjusted for age, gender, hemodialysis vintage, Charlson Comorbidity Index, and physical activity, and body mass index, in non-DM patients, IR was significantly linked with higher odds of low HDL-C (OR, 2.14, 95%CI, 1.23-3.75, p < .01), high TG (OR, 3.22, 95%CI, 1.69-6.12, p < .001), IFG (OR, 12.54, 95%CI, 6.39-24.63, p < .001), and hypoalbuminemia (OR, 6.59, 95%CI, 1.81-23.95, p <.01), but with lower odd of hyperkalemia (OR, 0.31, 95%CI, 0.17-0.57, p < .001). In DM patients, IR was significantly linked with higher odds of low HDL-C (OR, 3.07, 95%CI, 1.28-7.33, p < .05) and high TG (OR, 4.29, 95%CI, 2.05-8.98, p < .001). In overall sample, the elevated level of HOMA-IR was significantly associated with higher odds of low HDL-C (OR, 2.53, 95%CI, 1.59-4.01, p <.001), high TG (OR, 3.58, 95%CI, 2.25-5.69, p < .001), IFG (OR, 7.99, 95%CI, 4.50-14.18, p < .001), and hypoalbuminemia (OR, 3.07, 95%CI, 1.51-6.23, p <.01), but with lower odd of hyperkalemia (OR, 0.56, 95%CI, 0.36-0.88, p < .05; Tables 2 and 3).

## 4. Discussion

The level of IR is higher in overweight/obese and DM patients than in normal weight and non-DM patients in the current study. Obesity is also reported as the most common cause of IR previously [7].

In regard to traditional CVD risks, IR is significantly associated with a higher prevalence of dyslipidemia such as low HDL-C, high TG in the current study. The finding was also found in both the general population [43] and hemodialysis patients [9]. In the previous study, IR was found to reduce HDL-C in hemodialysis patients [9]. On the other hand, IR was also associated with higher likelihood of having impaired fasting glucose in all groups of patients, independent of age, gender, hemodialysis vintage, Charlson comorbidity index, Physical activity, and body mass index. The IR was well-documented as the immediate factor between obesity and impaired fasting glucose and cardiovascular diseases in the literature [5, 8]. Therefore, early interventions at stages of obesity or IR are extremely important to prevent and mitigate the adverse consequences of CVD risks, such as dietary intake, physical activities, and medication [5].

Regarding the nontraditional risks, the prevalence of elevated hs-CRP was higher in overweight/obese patients than those with normal weight. A previous study has also reported that patients with central obesity had higher hs-CRP level than those without [44]. The level of hs-CRP was not significantly differed among HD patients with and without DM. This was also found in HD patients in Japan that hs-CRP was similar between HD patients with DM, HD patients with metabolic syndrome (MS), and those with neither DM nor MS [9]. In addition, the prevalence of malnutrition in the current study was with 12.0% hypoalbumin; it is much lower than in the previous study conducted in Turkey with 44.1% hypoalbumin [45].

In the current study, there is also no significant association between IR and hs-CRP in patients with DM or non-DM patients or overweight/obese, but it existed in normal weight patients. The association between IR and hs-CRP was found in the overall sample in Turkey [11, 12] and in 598 overweight/obese patients in Spain [44]. On the other hand, the association between IR and hypoalbumin was found in hemodialysis patients with normal weight, and non-DM, and overall sample. This was also shown in a previous study [12]. It is important to take into account the evaluation of IR level, hs-CRP, and serum albumin, in order to prevent and intervene against CVD risks and diseases.

There were some contradictory findings between the current study and the previous one. Firstly, the IR associated with a lower likelihood of having high SBP in the current study which was in contrast with the previous finding [10]. Next, IR was related to a lower likelihood of hyperkalemia in normal weight, non-DM, and overall sample. This happened as the result of kalemia lowering treatment among hemodialysis patients. Finally, in normal weight, and non-DM sample, IR was associated with a lower likelihood of hyperphosphatemia in the current study. Hyperphosphatemia and high level of CaxPO_4_ combination were found to link with higher all-cause mortality in hemodialysis patients [46].

The study was of a cross-sectional nature; the causal relationship, therefore, cannot be generated. The interpretation of results should be cautious. The data related to supplement intake and medication was not explored. Therefore, the association between IR and some CVD risks was not well explained. Smoking is known as a major traditional cardiovascular risk factor. It is reported that 85.1% of patients were nonsmokers [47]. In the United State, based on data of USRDS, 6.2% of dialysis patients were smokers [48]. Therefore, we did not collect the data on smoking status in the current study. Future longitudinal, case-control, or intervention studies are encouraged to carefully examine the association.

## 5. Conclusions

The insulin resistance (IR) and CVD risks were common in hemodialysis patients. IR was associated with a higher prevalence of dyslipidemia (low HDL-C, high TG), impaired fasting glucose, elevated hs-CRP, and hypoalbuminemia. Addressing the assessment and treatment of IR and CVD risks in clinical practice could help with improving the hemodialysis outcomes.

## Figures and Tables

**Table 1:** Characteristics of hemodialysis patients.

	Total sample (n=384)	BMI < 24.0 (n=229)	BMI ≥ 24.0 (n=155)	p value	Non-DM (n=230)	DM (n=154)	p value

Characteristics							
Age, years	60.9 ± 11.8	61.0 ± 12.0	60.7 ± 11.5	0.809	59.4 ± 11.9	63.0 ± 11.2	0.003
Gender	223 (58.1%)	127 (55.5%)	96 (61.9%)	0.207	129 (56.1%)	94 (61.0%)	0.335
Dialysis vintage, years	5.5 ± 4.9	6.1 ± 5.4	4.7 ± 4.0	0.007	6.8 ± 5.6	3.7 ± 2.8	<0.001
CCI score	4.7 ± 1.6	4.6 ± 1.6	4.8 ± 1.5	0.295	4.1 ± 1.4	5.5 ± 1.3	<0.001
PA, MET-min/wk	4911.6 ± 1871.8	4874.5 ± 1900.3	4966.5 ± 1833.7	0.637	4964.7 ± 1929.0	4832.4 ± 1786.3	0.498
BMI, kg/m^2^	23.5 ± 3.9	21.0 ± 1.9	27.2 ± 3.0	<0.001	22.6 ± 3.4	24.9 ± 4.1	<0.001
DM	154 (40.1%)	66 (28.8%)	88 (56.8%)	<0.001	-	-	-
HTN	191 (49.7%)	107 (46.7%)	84 (54.2%)	0.151	108 (47.0%)	83 (53.9%)	0.183
CVD	115 (29.9%)	63 (27.5%)	52 (33.5%)	0.205	55 (23.9%)	60 (39.0%)	0.002
Traditional CVD risks							
SBP ≥ 130 mmHg	318 (82.8%)	189 (82.5%)	129 (83.2%)	0.860	181 (78.7%)	137 (89.0%)	0.009
DBP ≥ 85 mmHg	98 (25.5%)	60 (26.2%)	38 (24.5%)	0.710	59 (25.7%)	39 (25.3%)	0.942
TC ≥ 200 mg/dL	64 (16.7%)	33 (14.4%)	31 (20.0%)	0.149	38 (16.5%)	26 (16.9%)	0.926
LDL-C ≥ 100 mg/dL	186 (48.4%)	39 (17.0%)	32 (20.6%)	0.371	45 (19.6%)	26 (16.9%)	0.507
HDL-C < 40 mg/dL for men, < 50 mg/dL for women	253 (65.9%)	133 (58.1%)	120 (77.4%)	<0.001	132 (57.4%)	121 (78.6%)	<0.001
TG ≥ 150 mg/dL	156 (40.6%)	68 (29.7%)	88 (56.8%)	<0.001	68 (29.6%)	88 (57.1%)	<0.001
IFG (FPG ≥ 100 mg/dL, or DM)	267 (69.5%)	145 (63.3%)	122 (78.7%)	0.001	113 (49.1%)	154 (100.0%)	<0.001
Non-traditional CVD risks							
Hemoglobin < 11 g/dL	224 (58.3%)	137 (59.8%)	87 (56.1%)	0.471	131 (57.0%)	93 (60.4%)	0.504
Calcium < 8.4 mg/dL	32 (8.3%)	17 (7.4%)	15 (9.7%)	0.017	18 (7.8%)	14 (9.1%)	0.906
Calcium > 9.5 mg/dL	135 (35.2%)	69 (30.1%)	66 (42.6%)		81 (35.2%)	54 (35.1%)	
Phosphate < 3.5 mg/dL	27 (7.0%)	16 (7.0%)	11 (7.1%)	0.079	16 (7.0%)	11 (7.1%)	0.992
Phosphate > 5.5 mg/dL	136 (35.4%)	71 (31.0%)	65 (41.9%)		82 (35.7%)	54 (35.1%)	
Ca x PO_4_ ≥ 55 mg^2^/dL^2^	98 (25.5%)	45 (19.7%)	53 (34.2%)	0.001	58 (25.2%)	40 (26.0%)	0.868
i-PTH ≥ 300 pg/mL	164 (42.7%)	99 (43.2%)	65 (41.9%)	0.801	111 (48.3%)	53 (34.4%)	0.007
Hcy > 14 *μ*mol/L	329 (85.7%)	196 (59.6%)	133 (40.4%)	0.953	195 (59.3%)	134 (40.7%)	0.541
hs-CRP > 0.3 mg/L	175 (45.6%)	59 (25.8%)	55 (35.5%)	0.041	60 (26.1%)	54 (35.1%)	0.059
Albumin ≤ 3.5 g/dL	46 (12.0%)	26 (11.4%)	20 (12.9%)	0.646	19 (8.3%)	27 (17.5%)	0.006
Serum K ≥ 5.0 mEq/L	135 (35.2%)	82 (35.8%)	53 (34.2%)	0.745	88 (38.3%)	47 (30.5%)	0.119
HOMA-IR	5.4 (2.2, 11.0)	3.9 (1.7, 9.8)	6.9 (3.8, 14.3)	<0.001	4.1 (1.8, 9.9)	6.4 (3.3, 12.5)	<0.001
Others							
Pre-BUN, mg/dL	72.5 ± 19.9	72.6 ± 19.6	72.3 ± 20.5	0.870	73.9 ± 20.7	70.4 ± 18.6	0.090
Post-BUN, mg/dL	20.0 ± 10.4	19.2 ± 12.0	21.2 ± 7.2	0.066	19.6 ± 7.6	20.6 ± 13.5	0.362
Uric acid, mg/dL	7.3 ± 1.3	7.1 ± 1.2	7.5 ± 1.4	0.002	7.4 ± 1.2	7.0 ± 1.3	0.003
Creatinine, mg/dL	11.1 ± 2.2	10.7 ± 1.8	11.7 ± 2.4	<0.001	11.4 ± 2.0	10.6 ± 2.2	0.001
FPG, mg/dL	132.4 ± 58.1	127.2 ± 59.8	139.9 ± 54.9	.036	113.0 ± 41.7	161.3 ± 66.7	<0.001
FPI, *μ*U/mL	17.2 (8.8, 32.0)	14.1 (6.8, 27.0)	23.0 (12.7, 36.8)	<0.001	15.5 (7.6, 32.4)	19.2 (9.7, 31.6)	0.283

Categorical data is shown as n (%). Continuous data is presented as mean ± SD or median (interquartile range). P values calculated using Chi-square test, independent-samples T test, or Mann-Whitney *U* test.

^a^ The diagnosed values were defined by the National Kidney Foundation Kidney Disease Outcomes Quality Initiative Work Group.

BMI, body mass index; DM, diabetes mellitus; HTN, hypertension; CVD, cardiovascular diseases; CCI, Charlson comorbidity index; PA, physical activity; MET, metabolic equivalent minute/ week; SBP, systolic blood pressure; DBP, diastolic blood pressure; TC, total cholesterol; LDL-C, low-density lipoprotein cholesterol; HDL-C, high-density lipoprotein cholesterol; TG, triglyceride; IFG, impaired fasting glucose; CaxPO4, calcium phosphate product; iPTH, intact parathyroid hormone; Hcy, homocysteine; hs-CRP, high sensitive C-reactive protein; K, serum potassium; HOMA-IR, homeostatic model assessment of insulin resistance; BUN, blood urea nitrogen; FPG, fasting plasma glucose; FPI: fasting plasma insulin.

**Table 2:** Odd ratios of having traditional cardiovascular risks among hemodialysis patients with insulin resistance.

	High SBP	High DBP	High TC	High LDL-C	Low HDL-C	High TG	IFG
	OR (95%CI)	OR (95%CI)	OR (95%CI)	OR (95%CI)	OR (95%CI)	OR (95%CI)	OR (95%CI)
Normal weight							
Model 1	0.42 (0.20, 0.86)^*∗*^	0.90 (0.50, 1.63)	0.80 (0.38, 1.68)	0.83 (0.49, 1.39)	1.68 (0.99, 2.86)	2.79 (1.54, 5.06)^*∗∗*^	8.22 (4.35, 15.52)^*∗∗∗*^
Model 2	0.36 (0.17, 0.76)^*∗∗*^	1.13 (0.60, 2.12)	0.81 (0.37, 1.75)	0.78 (0.45, 1.36)	1.75 (1.01, 3.06)^*∗*^	3.41 (1.78, 6.53)^*∗∗∗*^	8.15 (4.14, 16.02)^*∗∗∗*^
Overweight/Obese							
Model 1	1.68 (0.71, 3.97)	1.05 (0.51, 2.19)	1.14 (0.52, 2.50)	0.84 (0.45, 1.58)	3.65 (1.58, 8.45)^*∗∗*^	2.90 (1.50, 5.61)^*∗∗*^	6.06 (2.33, 15.74)^*∗∗∗*^
Model 2	1.69 (0.66, 4.29)	0.75 (0.33, 1.68)	0.99 (0.42, 2.33)	0.80 (0.41, 1.55)	4.15 (1.71, 10.06)^*∗∗*^	3.06 (1.53, 6.13)^*∗∗*^	10.76 (3.36, 34.5)^*∗∗∗*^
Non-diabetes							
Model 1	0.69 (0.37, 1.31)	1.15 (0.63, 2.07)	0.78 (0.39, 1.56)	0.90 (0.54, 1.51)	2.38 (1.39, 4.07)^*∗∗*^	3.40 (1.85, 6.25)^*∗∗∗*^	10.64 (5.78, 19.58)^*∗∗∗*^
Model 2	0.61 (0.31, 1.18)	1.21 (0.64, 2.30)	0.64 (0.31, 1.35)	0.76 (0.44, 1.33)	2.14 (1.23, 3.75)^*∗∗*^	3.22 (1.69, 6.12)^*∗∗∗*^	12.54 (6.39, 24.63)^*∗∗∗*^
Diabetes							
Model 1	1.49 (0.54, 4.15)	0.93 (0.45, 1.93)	1.00 (0.43, 2.32)	0.77 (0.41, 1.45)	2.40 (1.07, 5.38)^*∗*^	3.75 (1.91, 7.37)^*∗∗∗*^	
Model 2	2.11 (0.70, 6.31)	0.94 (0.43, 2.05)	1.16 (0.44, 3.05)	0.78 (0.40, 1.54)	3.07 (1.28, 7.33)^*∗*^	4.29 (2.05, 8.98)^*∗∗∗*^	
Overall							
Model 1	0.80 (0.47, 1.37)	1.00 (0.63, 1.58)	0.80 (0.47, 1.37)	0.72 (0.48, 1.07)	2.65 (1.71, 4.11)^*∗∗∗*^	3.67 (2.39, 5.66)^*∗∗∗*^	8.14 (4.77, 13.89)^*∗∗∗*^
Model 2	0.76 (0.43, 1.32)	1.11 (0.68, 1.83)	0.75 (0.42, 1.32)	0.63 (0.41, 0.96)^*∗*^	2.53 (1.59, 4.01)^*∗∗∗*^	3.58 (2.25, 5.69)^*∗∗∗*^	7.99 (4.50, 14.18)^*∗∗∗*^

Significant level at ^*∗*^ p < 0.05, ^*∗∗*^ p < 0.01, and ^*∗∗∗*^ p < 0.001.

Model 1: elevated HOMA-IR and traditional CVD risk factors.

Model 2: adjusted for age, gender, hemodialysis vintage, Charlson comorbidity index, physical activity, and body mass index (for nondiabetes, diabetes, and overall sample).

SBP, systolic blood pressure; DBP, diastolic blood pressure; TC, total cholesterol; LDL-C, low-density lipoprotein cholesterol; HDL-C, high-density lipoprotein cholesterol; TG, triglyceride; IFG, impaired fasting glucose.

**Table 3:** Odd ratios of having nontraditional cardiovascular risks among hemodialysis patients with insulin resistance.

	Anemia	Low Ca	High Ca	Low PO4	High PO4	High CaxPO4	High iPTH	High Hcy	High CRP	Low Alb	Hyperkalemia
	OR (95%CI)	OR (95%CI)	OR (95%CI)	OR (95%CI)	OR (95%CI)	OR (95%CI)	OR (95%CI)	OR (95%CI)	OR (95%CI)	OR (95%CI)	OR (95%CI)
Normal weight											
Model 1	1.17 (0.69, 1.99)	0.99 (0.36, 2.72)	0.68 (0.38, 1.21)	1.86 (0.61, 5.62)	0.52 (0.29, 0.93)^*∗*^	0.47 (0.24, 0.93)^*∗*^	0.77 (0.45, 1.30)	1.08 (0.52, 2.27)	2.19 (1.18, 4.03)^*∗*^	9.25 (2.69, 31.79)^*∗∗∗*^	0.39 (0.22, 0.68)^*∗∗*^
Model 2	0.98 (0.55, 1.72)	1.25 (0.42, 3.69)	0.72 (0.39, 1.32)	1.71 (0.54, 5.40)	0.52 (0.28, 0.96)^*∗*^	0.51 (0.25, 1.03)	0.83 (0.48, 1.44)	0.98 (0.45, 2.12)	2.21 (1.16, 4.22)^*∗*^	8.31 (2.35, 29.37)^*∗∗*^	0.39 (0.22, 0.70)^*∗∗*^
Overweight/Obese											
Model 1	0.84 (0.45, 1.59)	4.27 (1.24, 14.69)^*∗*^	1.86 (0.95, 3.65)	3.72 (0.92, 15.08)	1.63 (0.84, 3.15)	1.26 (0.65, 2.45)	1.39 (0.74, 2.65)	0.96 (0.39, 2.36)	0.80 (0.41, 1.55)	0.83 (0.32, 2.13)	0.71 (0.36, 1.38)
Model 2	0.82 (0.41, 1.62)	3.00 (0.80, 11.20)	1.94 (0.96, 3.90)	3.54 (0.84, 14.96)	1.69 (0.82, 3.51)	1.23 (0.60, 2.50)	1.36 (0.68, 2.71)	0.98 (0.38, 2.53)	1.04 (0.51, 2.10)	0.68 (0.25, 1.82)	0.87 (0.42, 1.79)
Non-diabetes											
Model 1	1.04 (0.62, 1.75)	1.55 (0.57, 4.24)	0.87 (0.50, 1.52)	2.74 (0.84, 8.93)	0.65 (0.37, 1.13)	0.83 (0.46, 1.51)	0.97 (0.58, 1.62)	1.07 (0.52, 2.20)	1.58 (0.87, 2.86)	6.03 (1.71, 21.32)^*∗∗*^	0.32 (0.18, 0.56)^*∗∗∗*^
Model 2	1.04 (0.60, 1.82)	1.44 (0.49, 4.20)	0.75 (0.42, 1.36)	2.62 (0.78, 8.85)	0.51 (0.27, 0.95)^*∗*^	0.62 (0.32, 1.22)	0.86 (0.50, 1.48)	1.01 (0.47, 2.16)	1.58 (0.85, 2.95)	6.59 (1.81, 23.95)^*∗∗*^	0.31 (0.17, 0.57)^*∗∗∗*^
Diabetes											
Model 1	1.18 (0.62, 2.25)	2.38 (0.74, 7.71)	1.79 (0.90, 3.55)	2.24 (0.61, 8.22)	1.73 (0.87, 3.42)	1.00 (0.49, 2.06)	1.50 (0.77, 2.93)	0.63 (0.24, 1.64)	1.26 (0.65, 2.44)	2.31 (0.96, 5.52)	1.06 (0.54, 2.11)
Model 2	1.12 (0.56, 2.21)	2.11 (0.55, 8.15)	1.59 (0.77, 3.28)	2.41 (0.62, 9.40)	1.70 (0.82, 3.49)	0.97 (0.45, 2.06)	1.71 (0.82, 3.54)	0.74 (0.27, 2.04)	1.22 (0.60, 2.48)	2.05 (0.80, 5.26)	1.18 (0.57, 2.46)
Overall											
Model 1	1.09 (0.73, 1.64)	1.53 (0.72, 3.25)	1.03 (0.67, 1.59)	2.53 (1.06, 6.02)^*∗*^	1.01 (0.66, 1.54)	0.95 (0.60, 1.50)	0.88 (0.59, 1.32)	1.24 (0.70, 2.19)	1.42 (0.91, 2.21)	3.23 (1.62, 6.45)^*∗∗*^	0.51 (0.33, 0.78)^*∗∗*^
Model 2	1.06 (0.69, 1.63)	1.38 (0.61, 3.15)	0.95 (0.60, 1.49)	2.35 (0.97, 5.71)	0.99 (0.62, 1.57)	0.89 (0.54, 1.46)	0.93 (0.61, 1.43)	1.27 (0.70, 2.30)	1.30 (0.82, 2.06)	3.07 (1.51, 6.23)^*∗∗*^	0.56 (0.36, 0.88)^*∗*^

Significant level at ^*∗*^ p < 0.05, ^*∗∗*^ p < 0.01, ^*∗∗∗*^ p < 0.001.

Model 1: elevated HOMA-IR and traditional CVD risk factors.

Model 2: adjusted for age, gender, hemodialysis vintage, Charlson comorbidity index, physical activity, and body mass index (for nondiabetes, diabetes, and overall sample).

Ca, serum calcium; PO_4_, serum phosphorus; CaxPO4, calcium phosphorus product; iPTH, intact parathyroid hormone; hs-CRP, high sensitive C-reactive protein; Alb, serum albumin.

## Data Availability

Since the dataset contains sensitive and identifying information, any modification or deidentification on the dataset is restricted. The authors confirm that the data is available upon request. Requests may be sent to the corresponding author, Shwu-Huey Yang (sherry@tmu.edu.tw).
